# One-Week Self-Guided Internet Cognitive Behavioral Treatments for Insomnia in Adults With Situational Insomnia During the COVID-19 Outbreak

**DOI:** 10.3389/fnins.2020.622749

**Published:** 2021-01-21

**Authors:** Chenxi Zhang, Lulu Yang, Shuai Liu, Yan Xu, Huirong Zheng, Bin Zhang

**Affiliations:** ^1^Department of Psychiatry, Nanfang Hospital, Southern Medical University, Guangzhou, China; ^2^Guangdong-Hong Kong-Macao Greater Bay Area Center for Brain Science and Brain-Inspired Intelligence, Guangzhou, China; ^3^Guangdong Mental Health Center, Guangdong Provincial People's Hospital, Guangdong Academy of Medical Sciences, Affliated School of Medicine of South China University of Technology, Guangzhou, China

**Keywords:** COVID-19, CBTI, insomnia, situational insomnia, sleep disorder

## Abstract

**Objective:** In the current global home confinement due to COVID-19, most individuals are facing unprecedented stress which can induce situational insomnia. We explored the efficacy of self-guided online cognitive behavioral treatment for insomnia (CBTI) on situational insomnia during the COVID-19 outbreak.

**Methods:** Participants were recruited from March to April in 2020 in Guangzhou, China. A 1-week Internet CBTI intervention was performed for all individuals with situational insomnia. The Pre-sleep Arousal Scale (PSAS), Insomnia Severity Index (ISI), and Hospital Anxiety and Depression Scale (HADS) were measured before and after the intervention and compared between individuals who completed the intervention and those who did not.

**Results:** One hundred and ninety-four individuals with situational insomnia were included. For PSAS score, significant group effects were found on total score (*p* = 0.003), somatic score (*p* = 0.014), and cognitive score (*p* = 0.009). Time effect was significant on total score (*p* = 0.004) and cognitive score (*p* < 0.001). There was a significant group × time effect of the somatic score (*p* = 0.025). For ISI total score, there were significant time effect (*p* < 0.001) and group × time effect (*p* = 0.024). For the HADS score, a significant group effect was found on the anxiety score (*p* = 0.045). The HADS had significant time effects for anxiety and depressive symptoms (all *p* < 0.001).

**Conclusion:** Our study suggests good efficacy of CBTI on situational insomnia during COVID-19 for adults in the community, as well as on pre-sleep somatic hyperarousal symptom. The CBTI intervention is not applied to improve pre-sleep cognitive hyperarousal, depression, and anxiety symptoms.

## Introduction

The coronavirus disease 2019 (COVID-19) has thrown the world into the mire. Countries are in a state of blockade, the economy has stalled, and many people worry about their health and that of their loved ones. In the current situation of global home confinement due to the outbreak of COVID-19, most individuals are facing unprecedented stressful pressures of unknown duration. This may even increase daytime stress, anxiety, and depression symptoms and may also disrupt sleep. Sleep is essential for physical health and effective functioning of the immune system. It is also a key contributing factor to emotional and mental health and helps to relieve stress, depression, and anxiety symptoms (Salari et al., 2020). In the face of the COVID-19 outbreak, sleep has a wide range of physical and mental health benefits, so it becomes more important (Altena et al., 2020).

Millions of people are suffering from insomnia even before the COVID-19 outbreak (Cao et al., 2017), and to make matters worse, COVID-19 has brought new challenges to people who had no previous sleep problems (Zhang et al., 2020). As a form of primary insomnia as indicated in the *Diagnostic and Statistical Manual of Mental Disorders* (DSM)-V, situational insomnia lasts no more than 1 month (Ellis et al., 2012). The 3P model of situational insomnia (predisposing, precipitating, and perpetuating), also called acute insomnia, was proposed by Spielman et al. (1987) in the 1980's. The stress diathesis of situational insomnia is conceptualized by pre-disposing and precipitating developments. The pre-disposing factors make individuals more susceptible to insomnia, which is then exacerbated by precipitating events, leading to the threshold of insomnia being exceeded and the beginning of continuous periodic sleep interruptions. Predisposing factors are biological social–psychological factors, and precipitating factors are developed by threats or stressful life events. The COVID-19 outbreak seems to be a precipitating factor for the development of situational insomnia in a stressful atmosphere. Stress-related sleep problems can induce situational insomnia (Altena et al., 2020), which can further develop into chronic insomnia with repeated bouts (Bonnet and Arand, 2003).

Various non-pharmacological treatments have been developed for insomnia (Van Straten et al., 2017). These non-pharmacological treatments can be classified as educational (psychoeducation and sleep hygiene), behavioral (relaxation, paradoxical intention, sleep restriction, and stimulus control), or cognitive (worrying excessively about sleep and identifying and challenging dysfunctional thoughts), which are commonly referred to as cognitive behavioral therapy for insomnia (CBTI). The efficacy of CBTI has been systematically reviewed and written in the meta-analysis for the treatment of primary insomnia (Van Straten et al., 2017). Therefore, the American College of Physicians recommends CBTI as the initial treatment for insomnia in all adults (Van Straten et al., 2017). However, little evidence has been found on the management for situational insomnia until now.

COVID-19 does not influence everyone in the same way. Confirmed cases and frontline medical staff face the direct impacts of COVID-19 (Kokou-Kpolou et al., 2020; Voitsidis et al., 2020; Zhang et al., 2020). However, as we have seen all over the world, the consequences have spread far and wide and have caused major obstacles to sleep. The general population has less possibility to attract enough attention and necessary treatment during the COVID-19 outbreak. To lower the negative impact on sleep among the general population, our study tried to perform self-guided online CBTI to the general population in a community with situational insomnia during the COVID-19 outbreak. Face-to-face CBTI was not applied due to the lockdown restriction under the pandemic. We hypothesized that CBTI could provide good improvement in sleep quality among adults with situational insomnia in the community during the COVID-19 pandemic.

## Methods

### Participants

Participants were recruited from March to April in 2020 via a campaign of the “Prevention and Protection Handbook Against Epidemic” supported by the local government. At the end of the handbook, a QR code is printed, and people can participate in the study voluntarily by scanning it through the WeChat program. Fifty thousand copies of the handbook were distributed to different communities in Guangzhou City, Guangdong Province, China. The COVID-19 pandemic in Guangdong province has been under control, and people have started to go back to work in late February. For example, on March 23rd, eight newly confirmed cases were reported with a total of 1,407 confirmed cases, including a total of eight deaths (News Guangdong, 2020). Guangzhou City has the second highest number of confirmed cases in Guangdong Province. A total of 280 subjects from the general population in the community scanned the QR code and registered. Some of them can be diagnosed with situational insomnia, and some cannot. There was no waiting list in our study. All the participants voluntarily logged in for the CBTI intervention. Finally, we summarized their login times and distributed them into different groups (completers group: login times = 7, non-completers group: login time ≤ 6). All the participants were suggested to log into their WeChat account to enter the CBTI applet of WeChat for 7 days. A pre-test online questionnaire was collected. Then, the online CBTI was offered for a week. After the first login, the system would record the login history and be closed automatically a week later. Then, the post-test online questionnaire was given, with a reminder that it be completed in 3 days. The participants were not compelled or persuaded to log into the CBTI applet of WeChat during the intervention. All the participants signed consent forms to continue at the beginning of the research. The participants who completed 7 days of login history in the CBTI applet of WeChat were allocated to the completers group, and those who did not would be in the non-completers group. For the 1-week CBTI, WeChat pushes core courses once a day, about 10–15 min each, including sleep hygiene education (day 1), sleep restriction (day 2), stimulation control (day 3), relaxation training (day 4), cognitive reconstruction (day 5), correct thoughts about sleeping medicine (day 6), and summary and review (day 7) (Yang et al., 2019).

**Figure 1:**
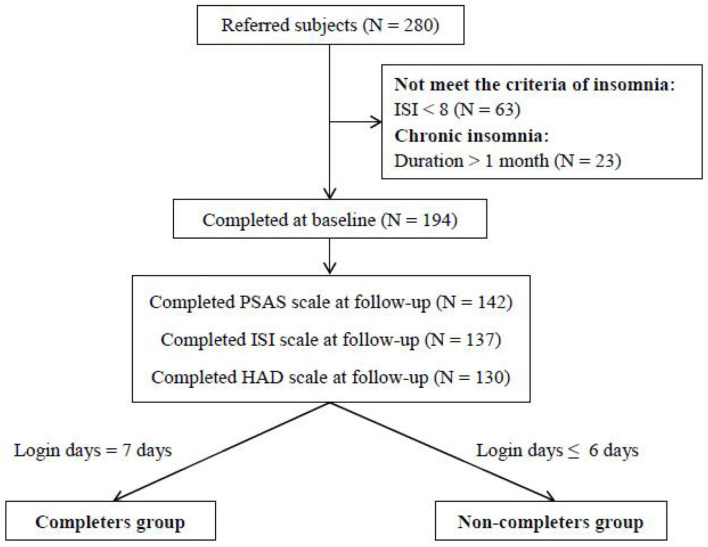
Flow chart of the study.

Inclusion criteria were as follows: (1) insomnia criteria according to DSM-V; (2) Insomnia Severity Index (ISI) score of 8 or higher; (3) insomnia duration of <1 month; and (4) age of 18 years or older. Of 280 participants, 63 and 23 were excluded for having an ISI <8 and insomnia lasting more than 1 month, respectively. The flow chart of this study is shown in Figure 1. Two hundred and eighty participants were referred in our study. The reasons for exclusions were as follows: (1) did not meet the criteria of insomnia (ISI < 8) (*N* = 63) and (2) were diagnosed with chronic insomnia (duration > 1 month) (*N* = 23). Finally, 194 individuals with situational insomnia were included in our study. There were no significant differences on the demographics of all participants (shown in Supplementary Table 1).

The study protocol was approved by the ethics committee of Nanfang Hospital, Southern Medical University.

### Measurement of Outcomes

The Pre-sleep Arousal Scale (PSAS) measures somatic and cognitive arousal right before sleep. The somatic/cognitive PSAS contains eight items and scores ranging from 8 to 40. Higher scores suggest a greater pre-sleep problem of somatic/cognitive arousal (Kalmbach et al., 2019). For the Chinese version of PSAS, the Cronbach α was 0.88 (Jan et al., 2009).

The ISI has seven items rated from 0 to 4 according to severity (Bastien et al., 2001). The ISI score measures initial, middle, and late insomnia; sleep satisfaction; interference of insomnia with daytime functioning; noticeability of sleep problems by others; and distress about sleep difficulties. The psychometric characteristics of ISI are adequate and sensitive to score change (Bastien et al., 2001), even when Internet based (Thorndike et al., 2011). For the Chinese version of the ISI, the Cronbach α was 0.81 (Yu, 2010).

The Hospital Anxiety and Depression Scale (HADS) has a seven-item anxiety subscale (HADS-A) and a seven-item depression subscale (HADS-D). Each item is scored from 0 to 3 (total subscale score range 0–21), with clinical severities of minimal (0–7), mild (8–10), moderate (11–13), and severe (14–21) depression or anxiety symptoms (Christensen et al., 2020). For the Chinese version of HADS, the Cronbach α was 0.86 for the full scale, 0.82 for the depression subscale, and 0.77 for the anxiety subscale (Leung et al., 1999).

### Statistical Analyses

All the analyses were performed with SPSS (version 21.0). Demographic data and sleep patterns were presented with mean, percentage, and interquartile range (IQR) as appropriate. Repeated-measures one-way ANOVA was conducted on all scales (PSAS, ISI, and HADS) with one within subject (time effect: baseline vs. follow-up) and one between subject (group effect: completer vs. non-completer). For those with a significant interaction effect (group × time effect), the change score was calculated as the difference between pre-test and post-test scores. Spearman correlations between change scores and demographic data were performed. All statistical significance was set at *p* = 0.05.

## Results

One hundred and ninety-four individuals with situational insomnia were included in our study. Table 1 shows the demographic data of individuals with situational insomnia who were 37 years old on average with a normal body mass index (BMI). The individuals were mostly female (70.1%), had a bachelor's degree (57.2%), were married (62.9%), were living with their children (44.3%), had a full-time job (76.8%), and earned 5,000–10,000 RMB monthly (30.4%). Table 2 presents the frequency of using tea, coffee, alcohol, and cigarette in individuals with situational insomnia. Majority of them seldom drank tea (33.0%), did not drink coffee (39.7%), did not drink alcohol (39.2%), and did not smoke (80.9%). Table 3 reflects the sleep patterns for individuals with situational insomnia at baseline. They often went to bed at 23:30 and woke up at 7:18 in the morning during the workday. For the weekend, the individuals went to sleep 18 min later at night and woke up 72 min later in the morning compared to the workday. Their sleep latency was more than 60 min (34.5%), 31–60 min (30.4%), 11–30 min (29.9%), and <10 min (5.2%). The average sleep duration was 6.5 h during the COVID-19 outbreak, and all of them (100%) thought their sleep was insufficient.

**Table 1:** Demographic data of individuals with situational insomnia at baseline.

	**Individuals with situational insomnia at baseline (*N* = 194)**
Age	37.1 ± 10.8
BMI	21.9 ± 3.1
Gender (female)	136 (70.1)
Education level	
High school or below	37 (19.1)
Bachelor degree	111 (57.2)
Graduate degree	46 (23.7)
Marital status	
Single	63 (32.5)
Married	122 (62.9)
Divorced	9 (4.6)
Living situation	
Alone	44 (22.7)
With parents	51 (26.3)
With child	86 (44.3)
With friends	13 (6.7)
Employment status	
Full time	149 (76.8)
Part time	6 (3.1)
Unemployed	11 (5.7)
Retired	15 (7.7)
Student	13 (6.7)
Monthly income	
<3,000 RMB	22 (11.3)
3,000–5,000 RMB	56 (28.9)
5,000–10,000 RMB	59 (30.4)
>10,000 RMB	57 (29.4)

**Table 2:** Usage of tea, coffee, alcohol, and cigarette in the past year among individuals with situational insomnia at baseline.

	**Individuals with situational insomnia at baseline (*N* = 194)**
Tea	
No	40 (20.6)
Seldom	64 (33.0)
Sometimes	37 (19.1)
Often (>3/week)	53 (27.3)
Coffee	
No	77 (39.7)
Seldom	62 (32.0)
Sometimes	30 (15.5)
Often (>3/week)	25 (12.9)
Alcohol	
No	76 (39.2)
Seldom	67 (34.5)
Sometimes	39 (20.1)
Often (>3/week)	12 (6.2)
Cigarette	
No	157 (80.9)
Seldom	14 (7.2)
Sometimes	9 (4.6)
Often (>3/week)	14 (7.2)

**Table 3:** Sleep pattern in individuals with situational insomnia at baseline.

	**Individuals with situational insomnia at baseline (*N* = 194)**
Time to go to bed (Monday–Friday) median (IQR)	23:30 (23:00–0:30)
Time to wake up (Monday–Friday) median (IQR)	7:18 (6:42–8:48)
Time to go to bed (weekend) median (IQR)	23:48 (23:00–0:12)
Time to wake up (weekend) median (IQR)	8:30 (7:30–10:00)
Daytime nap (min/day) median (IQR)	15.0 (0.0–40.0)
Sleep latency	
<10 min	10 (5.2)
11–30 min	58 (29.9)
31–60 min	59 (30.4)
>60 min	67 (34.5)
Sleep duration during COVID-19 (hour)	6.5 ± 3.1
Sufficient sleep (No)	194 (100.0)

**Figure 2:**
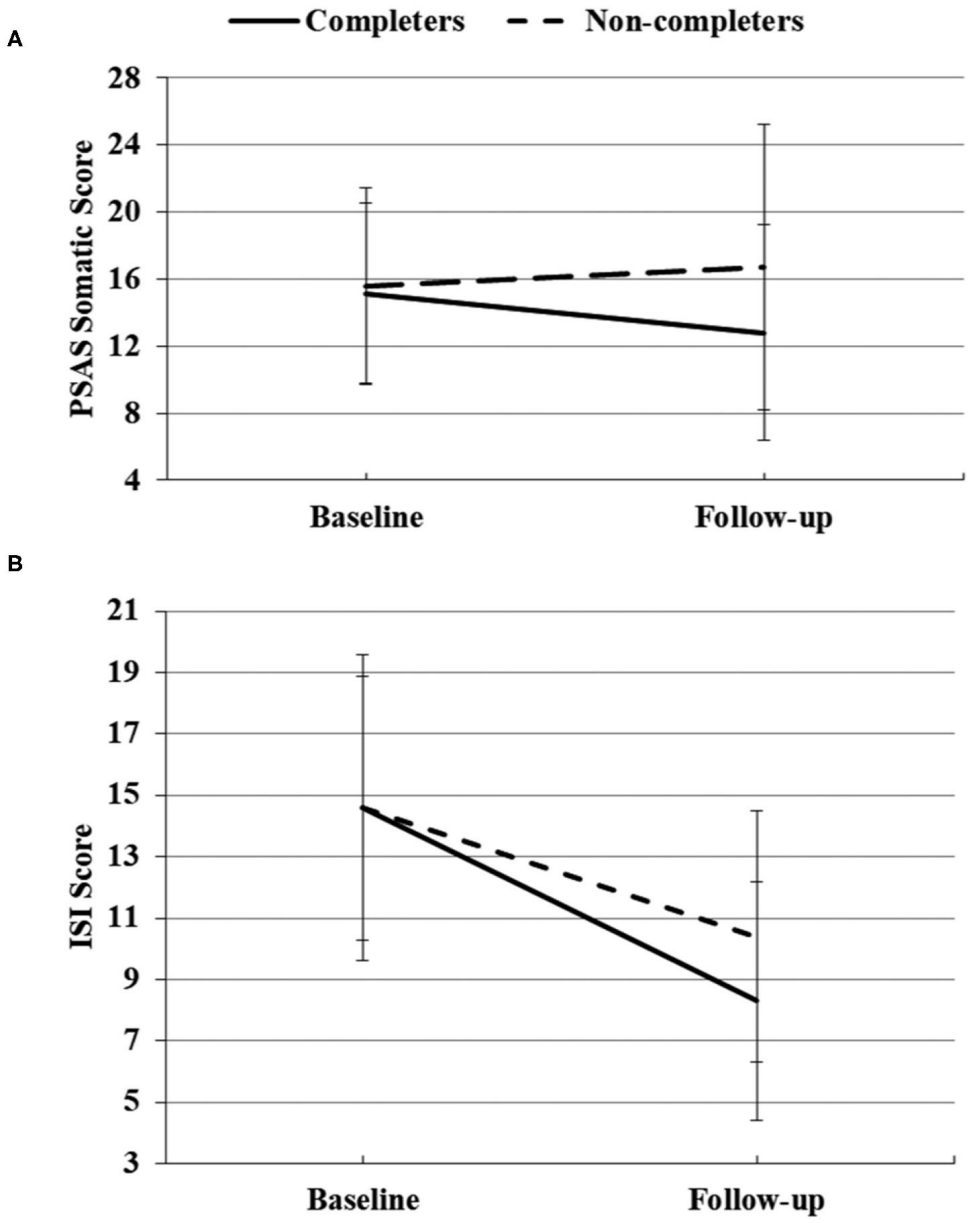
Significant group × time effects of PSAS somatic score **(A)** and ISI score **(B)** between completers and non-completers at baseline and follow-up.

At follow-up, the numbers of participants who completed PSAS, ISI, and HADS were 142, 137, and 130, respectively. Among them, 58 (40.8%) participants completed the CBTI for a week (7 days of login history) for the PSAS scale and 60 (43.8%) for the ISI scale and 55 (42.3%) for HAD scale. Table 4 shows the results of repeated-measures ANOVAs of measurements between the completers group and non-completers group from baseline to follow-up. For PSAS score, significant group effects were found for total score [*F*_(1, 140)_ = 8.856, *p* = 0.003, partial η^2^ = 0.059], somatic score [*F*_(1, 140)_ = 6.175, *p* = 0.014, partial η^2^ = 0.042], and cognitive score [*F*_(1, 140)_ = 7.049, *p* = 0.009, partial η^2^ = 0.048]. Time effect was significant for total score [*F*_(1, 140)_ = 8.805, *p* = 0.004, partial η^2^ = 0.059] and cognitive score [*F*_(1, 140)_ = 26.214, *p* < 0.001, partial η^2^ = 0.158]. There was a significant group × time effect for the somatic score [*F*_(1, 140)_ = 5.126, *p* = 0.025, partial η^2^ = 0.035) (Figure 2A). No significant differences were found for the rest of the PSAS scale. For ISI total score, there were significant time effect [*F*_(1, 135)_ = 128.487, *p* < 0.001, partial η^2^ = 0.488] and group × time effect [*F*_(1, 135)_ = 5.192, *p* = 0.024, partial η^2^ = 0.037] (Figure 2B). No significant group effect was found for the ISI score. For the HADS score, a significant group effect was found for the anxiety score [*F*_(1, 128)_ = 4.111, *p* = 0.045, partial η^2^ = 0.031). The HADS had significant time effects for the anxiety [*F*_(1, 128)_ = 15.441, *p* < 0.001, partial η^2^ = 0.108] and depressive symptoms [*F*_(1, 128)_ = 34.446, *p* < 0.001, partial η^2^ = 0.212]. There was no time × group interaction effect for the HADS.

**Table 4:** Repeated-measures ANOVAs of measurements between the completers group and non-completers group from baseline to follow-up.

	**Baseline**		**Follow-up**		**Group effect**	**Time effect**	**Group × time effect**
	**Completers**	**Non-completers**	**Completers**	**Non-completers**			
PSAS score							
Total	35.8 ± 9.6	38.1 ± 10.9	28.6 ± 13.0	36.5 ± 17.3	0.003	0.004	0.057
Somatic	15.1 ± 5.4	15.6 ± 5.8	12.8 ± 6.4	16.7 ± 8.5	0.014	0.418	0.025
Cognitive	20.7 ± 5.7	22.5 ± 6.9	15.8 ± 7.1	19.1 ± 9.1	0.009	<0.001	0.373
ISI total score	14.6 ± 4.3	14.6 ± 5.0	8.3 ± 3.9	10.4 ± 4.1	0.088	<0.001	0.024
HADS score							
Anxiety	7.8 ± 3.3	8.5 ± 4.8	5.7 ± 3.4	7.2 ± 3.5	0.045	<0.001	0.340
Depression	7.4 ± 3.9	7.6 ± 4.4	4.6 ± 3.0	5.5 ± 3.4	0.256	<0.001	0.463

**Figure 3:**
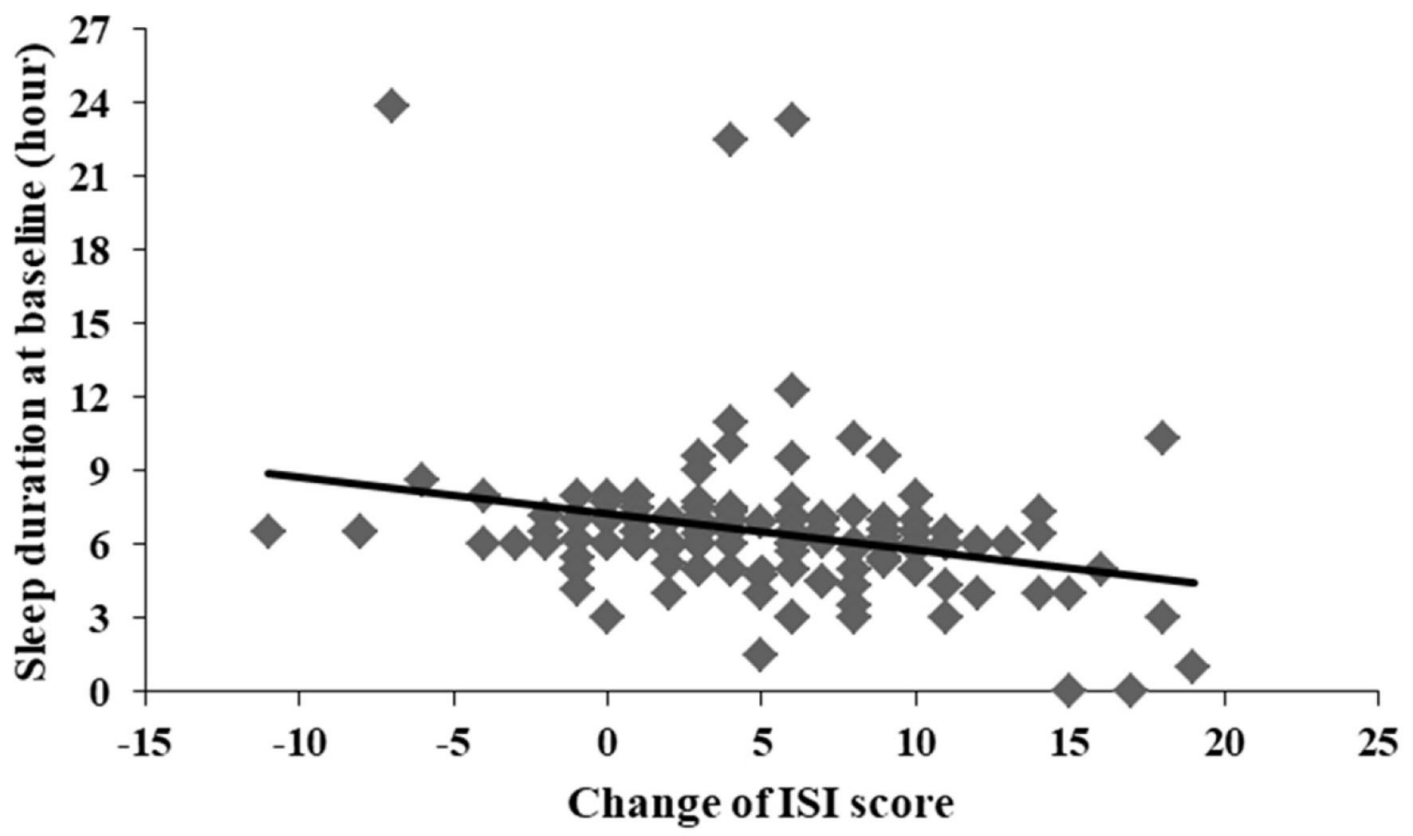
Significant correlation between change of ISI score and sleep duration at baseline (*r* = −0.267, *p* = 0.002).

A significant correlation was found between the change of ISI total score and sleep duration at baseline (*r* = −0.267, *p* = 0.002) (Figure 3). There were no significant differences between the change of ISI total score and left demographic data. No significant differences were observed between the change of PSAS somatic score and all demographic data.

## Discussion

In a sample of 194 Chinese participants, our study was first to investigate whether CBTI reduced situational insomnia symptoms among the general population in the community during the ongoing COVID-19 pandemic. More improvement in insomnia symptoms was correlated with shorter sleep duration at baseline. The participants who did not complete the 1-week CBTI showed more pre-sleep hyperarousal problems and more anxiety symptoms. At follow-up, reductions were found on pre-sleep cognitive hyperarousal, insomnia symptoms, depressive symptoms, and anxiety symptoms, except for pre-sleep somatic hyperarousal. The results of this study indicate that the treatment of CBTI also led to improvements in pre-sleep somatic hyperarousal. This finding appears to indicate CBTI was effective in treating pre-sleep somatic hyperarousal.

Consistent with previous studies testing CBTI on insomnia patients in community samples, the current study found that CBTI was highly effective at improving sleep among community individuals with situational insomnia during the COVID-19 outbreak (Talbot et al., 2014; Kaldo et al., 2015; Lancee et al., 2015, 2016; Norell-Clarke et al., 2015). There was no significant difference at baseline between participants who completed 1-week CBTI and those did not in our study, suggesting that the severity of insomnia before the intervention was the same. The baseline score of the ISI scale was relatively lower than that of other CBTI studies in community recruitment for chronic insomnia (Talbot et al., 2014; Kaldo et al., 2015; Lancee et al., 2015, 2016; Norell-Clarke et al., 2015) but consistent with a study of acute insomnia (Ellis et al., 2015). For online self-guided CBTI, a study of 30 participants with insomnia from a community in the Netherlands who accepted an 8-week CBTI intervention found that ISI scores (both pre-intervention and post-intervention) were higher than those in our study (Lancee et al., 2016). Another study of 148 insomnia patients from a community in Sweden showed a higher ISI score at baseline but a consistent ISI score after an 8-week CBTI intervention on chronic insomnia (Kaldo et al., 2015). One reason for the differences was that age and gender may play roles affecting insomnia. For example, the Netherlands' study had a sample that had an average age of 41.2 and was 86.7% female while Sweden's study had a sample that had a mean age of 47 and was 81% female. In our study, the mean age was 37.1, and the proportion of female gender was 70.1%, which indicated that we had younger and fewer female participants. As is known, older age and female gender were both related to more severe insomnia symptoms (Zhang and Wing, 2006; Cao et al., 2017). Another explanation for the deviation was that the insomniac individuals in our study all had situational insomnia while the above two studies included chronic insomnia patients. Before CBTI intervention, the present study was in line with the ISI score in a study of acute insomnia with 20 individuals (Ellis et al., 2015). In Ellis et al. (2015) study, the acute insomnia patients completed only a single session of CBTI for 1 h, indicating limited efficacy on insomnia symptoms. Our ISI score was lower, suggesting better improvement of insomnia symptoms, than that of Ellis et al. (2015) study. Our study also showed a correlation between change of ISI score and sleep duration at baseline. Individuals with shorter sleep duration at baseline were more likely to have better improvement of insomnia (better sleep). The improvement was high enough (shorter sleep duration at baseline and then longer sleep duration at follow-up) to be observed or screened by the test in a short duration.

CBTI was also efficient, when compared with the control, at reducing somatic hyperarousal before sleep. Somatic hyperarousal is a key feature of insomnia that maintains and perpetuates the cycle of poor sleep and daytime impairment (Bonnet and Arand, 1997; Harvey, 2002). The present study observed that CBTI can efficiently improve the pre-sleep somatic hyperarousal for individuals with insomnia, which was consistent with the previous study (Kalmbach et al., 2019). The PSAS somatic score was similar at baseline between the two groups (completers and non-completers). Then the PSAS somatic score decreased after CBTI intervention but increased in the control group, indicating a negative impact on pre-sleep somatic hyperarousal by COVID-19. Future longitudinal research with sampling at multiple time points could be used to test the causative relationships of COVID-19 and somatic hyperarousal problems.

No efficacies of CBTI were found on cognitive hyperarousal before sleep, depressive symptoms, or anxiety symptoms. More severe pre-sleep hyperarousal problems and anxiety symptoms were shown in the non-completers group, which indicated their worse compliance with the CBTI intervention accordingly. Previous studies have shown mixed results of the effect of CBTI on anxiety and depression (Espie et al., 2001; Belleville et al., 2007), while others have found no differences (Edinger et al., 2001; Jacobs et al., 2004; Rybarczyk et al., 2005; Taylor et al., 2018). One possible interpretation for the lack of variety in cognitive hyperarousal, anxiety, and depression in this study is that the two groups were both relatively healthy, reporting near-normal sleep patterns that produce a floor effect. There was no evidence of symptomatic depression (sample mean score = 7.8; the typical cutoff for the HADS-D is ≥8) or anxiety (sample mean score = 7.4; the typical cutoff for the HADS-A is ≥8) (Stern, 2014). Therefore, the non-significant changes in comorbid symptoms with treatment were likely due to a floor effect. On the other hand, the study populations were varied by age, gender, insomnia severity, and duration of insomnia. In our study, the participants were all individuals with situational insomnia who experienced a stronger negative impact on sleep due to the COVID-19 outbreak. Under the atmosphere of COVID-19, the stressful environment played an important role in the pre-sleep hyperarousal problems, depressive symptoms, and anxiety symptoms, which was different from other studies on chronic insomnia (Edinger et al., 2001; Jacobs et al., 2004; Rybarczyk et al., 2005; Taylor et al., 2018).

Situational insomnia may be due to several reasons. First, the home confinement, social distancing, school suspension, quarantines, and work from home all wrought profound changes to daily life routines. To adapt to a new daily schedule or lack of a normal schedule is often inconvenient. It was difficult to track time without typical time “anchors.” Staying at home for a long time may reduce light-based cues for wakefulness and sleep, known as zeitgebers, which are essential for the circadian rhythm (Pavlova, 2017). Second, worries were prevalent during the COVID-19 pandemic, which could be stressful. Naturally, people feared having the coronavirus or infecting other people unintentionally. Economic concerns were also affecting people. With the stagnation of the economy and the increase in unemployment, people usually worried about income, savings, and expenditures that should be kept within the limits of their income. The worries and anxiety about the unknowns persisted during the pandemic and disrupted sleep, including the spread of COVID-19, the hospital's ability to manage the epidemic, and the recovery time of the economy. Third, the epidemic brought about isolation, and the situation may be even worse for those with loved ones who were infected with or died from COVID-19. Home confinement aggravated misery and depression, both of which had the potential to induce sleep problems (Kim et al., 2009). Fourth, due to COVID-19, many families were stressed. The situation could be under pressure due to the canceled trips, isolation from friends, and an abundance of time at home. Obligations of work from home or management of children who were usually at school could pose real problems, generating stress and discord that have been shown to interrupt sleep (Kim and Dimsdale, 2007). Fifth, while working from home, social distancing increased screen time. Not only could it stimulate the brain, making the brain harder to relax, but the blue light from screens could also suppress the natural production of melatonin, a hormone that the body makes to help us sleep (Pilorz et al., 2016). Sixth, the chronic stress from a pandemic may induce physical symptoms, including persistent headaches and memory and digestive problems (Harris et al., 2017). Stress-related fatigue may also be another common side effect (Dinges, 2001). To sum it up, CBTI intervention effectively improved sleep quality during the COVID-19 outbreak by considering educational (psychoeducation and sleep hygiene), behavioral (relaxation, sleep restriction, stimulus control, and paradoxical intention), and cognitive (identifying and challenging dysfunctional thoughts and excessively worrying about sleep). When compared with traditional face-to-face CBTI, our study provided a better way to promote it to the general population during the COVID-19. Meanwhile, the self-guided Internet CBTI was helpful for the content control of CBTI and had the advantages of being simple, low in cost, and providing savings in manpower and space. The intervention could be accessed wherever there was an Internet network and was not impacted by isolation and home confinement.

### Limitation

There were several limitations in our study. First, the history of past sleep problems was not collected at baseline. We only evaluated acute sleep quality after the COVID-19 outbreak. Second, information about COVID-19, including any coronavirus infections or suspected fever cases among the participants and their family members; about living situations; and about other factors that may cause impressive stress was not asked. Third, our study was not able to confirm the causality relationship between COVID-19 and acute insomnia. Future studies with more time points are needed. Fourth, the study did not include the psychiatric history or current use of anxiolytic/sedative medication. Fifth, the screen tools in our study may not be sensitive enough for anxiety or depressive symptoms. Beck Inventory may be better than HADS. Sixth, a healthy control group was not included in our study. We used a group with bad compliance as a control group in our study.

## Conclusion

Our study suggests good efficacy of the self-guided Internet CBTI on situational insomnia during the COVID-19 outbreak for adults in the community, as well as on pre-sleep somatic hyperarousal symptom.

## Data Availability Statement

The raw data supporting the conclusions of this article will be made available by the authors, without undue reservation.

## Ethics Statement

The studies involving human participants were reviewed and approved by Institutional Review Board, Nanfang Hospital of Southern Medical University. The patients/participants provided their written informed consent to participate in this study.

## Author Contributions

BZ conceived and designed this study. CZ made additional contributions to its design. CZ conceived and conducted statistical analyses, with additional advice regarding analyses contributed by YX, LY, SL, and BZ. CZ drafted the manuscript. All authors contributed to editing it and approved the final manuscript.

## Conflict of Interest

The authors declare that the research was conducted in the absence of any commercial or financial relationships that could be construed as a potential conflict of interest.
