# Supplementary material for: One-Week Self-Guided Internet Cognitive Behavioral Treatments for Insomnia in Adults With Situational Insomnia During the COVID-19 Outbreak
Source: Front Neurosci. 2021 Jan 21;14:622749. doi: 10.3389/fnins.2020.622749 (PMC7859353; doi:10.3389/fnins.2020.622749)
Supplement: Supplementary file 1 [file Data_Sheet_1.PDF]

Table S1. Demographics of referred individuals

	Situational insomnia individuals at baseline (N = 194)	Healthy individuals (N = 63)	Chronic insomnia individuals (N = 23)	p[a]
Age	37.1 ± 10.8	35.5 ± 9.1	36.9 ± 10.4	0.568[b]
BMI index	21.9 ± 3.1	22.8 ± 3.2	21.8 ± 3.2	0.130[b]
Gender (female)	136 (70.1)	44 (69.8)	12 (52.2)	0.209
Education level				0.201
High school or below	37 (19.1)	4 (6.4)	3 (12.9)	
Bachelor degree	111 (57.2)	37 (58.7)	16 (69.6)	
Graduate degree	46 (23.7)	22 (34.9)	4 (17.3)	
Marital status				0.311
Single	63 (32.5)	29 (46.0)	6 (26.1)	
Married	122 (62.9)	32 (50.8)	16 (69.6)	
Divorced	9 (4.6)	2 (3.2)	1 (4.3)	
Living situation				0.209
Alone	44 (22.7)	16 (25.4)	5 (21.7)	
With parents	51 (26.3)	10 (15.9)	6 (26.1)	
With child	86 (44.3)	26 (41.3)	10 (43.5)	
With friends	13 (6.7)	11 (17.5)	2 (8.7)	
Employment status				0.188[c]

Full time	149 (76.8)	51 (81.0)	17 (73.9)	
Part time	6 (3.1)	5 (7.9)	0 (0)	
Unemployed	11 (5.7)	0 (0)	3 (13.0)	
Retired	15 (7.7)	3 (4.8)	1 (4.3)	
Student	13 (6.7)	4 (6.3)	2 (8.7)	
Monthly income				0.249
< 3,000 RMB	22 (11.3)	11 (17.5)	5 (21.7)	
3,000 – 5,000 RMB	56 (28.9)	13 (10.6)	2 (8.7)	
5,000 – 10,000 RMB	59 (30.4)	17 (27.0)	9 (39.1)	
> 10,000 RMB	57 (29.4)	22 (34.9)	7 (30.4)	

a ANOVA

b Chi-square test

c Fisher exact test
